# Left atrial appendage inversion: First computational study to shed light on the phenomenon

**DOI:** 10.1016/j.heliyon.2024.e26629

**Published:** 2024-02-23

**Authors:** Danila Vella, Giulio Musotto, Andrew Cook, Giorgia Maria Bosi, Gaetano Burriesci

**Affiliations:** a Bioengineering Group, Ri.MED Foundation, Palermo, Italy; b Department of Engineering, University of Palermo, Italy; c UCL Institute of Cardiovascular Science & Great Ormond Street Hospital for Children, London, United Kingdom; d UCL Mechanical Engineering, University College London, London, United Kingdom

**Keywords:** LAA inversion, Finite element analysis, LAA morphology, LAA prolapse, LAA eversion

## Abstract

Inversion of the left atrial appendage is a rare phenomenon, which may occur during the de-airing maneuvers associated to routinely performed surgery procedures, such as cardiopulmonary bypass or left ventricular assist device implantation. In this case, the body of the inverted appendage can obstruct the mitral valve leading to severe complications. The mechanisms are still poorly known, and more specific studies are needed to better understand its causes and identify mitigating strategies. The current study attempts to gain a better comprehension of the conditions and the factors favourable to left atrial appendage inversion. Four patient specific appendage morphologies, obtained from computerised tomography and representative of the main typologies commonly used for the appendage classification (chicken wing, cactus, cauliflower, and windsock), were used for the study. The numerical models were subjected to the same loading pattern, made of subsequent different pressure curves.

Results show that the morphologies invert and recover their original anatomical configuration at different pressure loads, indicating that their tendency to invert is associated to their specific morphological features. Moreover, the analysis highlights that, although restoring the physiological left atrium pressure is not sufficient to induce appendage recovery, pressures well below the ventricular ones can induce the return to the natural configuration.

All models recovered the anatomical configuration at pressures well below the ventricular pressure (about 100 mmHg), suggesting that basic trans-catheter maneuvers, e.g. producing temporary mitral regurgitation, could be attempted to correct the appendage configuration, prior to opt for more invasive surgical approaches.

## 1 Introduction

Left atrial appendage (LAA) is a sac of muscle tissue protruding from the left atrium. Recently, many studies focused on LAA, as it is anatomically prone to blood stasis, due to its tubular structure connected with the atrium by a narrow junction. In the presence of cardiac pathologies, such as atrial fibrillation, paroxysmal supraventricular tachycardia and atrial fibrosis, LAA represents a potential site for thrombus formation [1], [2], [3].

Being in continuity with the atrium, the LAA is designed to withstand the physiological atrial pressure variations set by the cardiac cycle. Temporary hemodynamics alterations due to clinical procedures could set abnormal atrial pressures, drawing the LAA back towards the left atrium until its body protrudes into the atrium cavity. This LAA inversion is reported as a rare clinical event, usually consequence of the suction produced in the atrium during the de-airing maneuvers or the vent performed after cardiac surgery, or as a result of the pressure unbalance resulting from Cardiopulmonary Bypass (CPB) or Left Ventricular Assist Device (LVAD) implantation [4], [5], [6], [7], [8], [9], [10], [11], [12], [13], [14], [15], [16], [17], [18], [19], [20], [21], [22], [23], [24], [25], [26], [27]. Moreover, this phenomenon was also observed during transcatheter LAA closure device placement, and is described as a potential complication for the device recapturing [28]. The inverted appendage, commonly mistaken for a mass, thrombus or vegetation, can lead to restricted inflow to the left ventricle, pulmonary hypertension, risks of embolic stroke, necrosis and/or hemodynamic disturbance [6], [8], [12], [15], [20], [26]. Hence, it is crucial that clinicians are aware of the risk of LAA inversion, so as to be prepared to a timely intervention to restore the natural LAA configuration and to avoid unnecessary therapies and plan the safest and most appropriate action [17], [18], [24].

Moreover, in recent years, partial LAA inversion has been suggested as a potential minimally invasive procedure for LAA exclusion [29], [30]. In particular, *Kassab* method consists in constraining the inverted distal portion inner the appendage walls. Similarly, *Sternil* method consists in hooking the inner tip with a catheter, using vacuum, to draw back the appendage into the left atrium and then ligating to occlude it. Since these methods represent a potential for future clinical procedures to prevent clot formation, an increasing interest will be dedicated to computational studies to investigate under which conditions LAA inversion establishes [31].

In this context, a better comprehension of the conditions and factors favourable to LAA inversion would be helpful to improve the clinical practice. For example, the morphological features of LAA, such as size of the atrial orifice or appendage height, might play a role in the LAA inversion [7], [14]. Moreover, LAA is characterised by highly variable morphologies, and it is therefore essential to understand if the specific anatomical features play a role in favouring LAA inversion. This work aims to improve the understanding of the factors which contribute to LAA inversion by means of computational structural analyses, and verify if computational simulations based on patients scans (e.g. computerized tomography imaging) performed prior to LVAD﹨CBP intervention could help to predict the risk of procedural LAA inversion and plan the maneuvers to be performed to restore the anatomical configuration. To achieve these aims, four patient specific numerical models, belonging to the different morphological classes based on the current classification [32], [33], were subjected to the same variations of pressure. The suction threshold determining the LAA inversion and the pressure conditions (physiological or unphysiological) leading to the recovering of their native configuration were estimated for each model, verifying the role of the patient specific morphology on the phenomenon.

## 2 Methods

**Figure 1:**
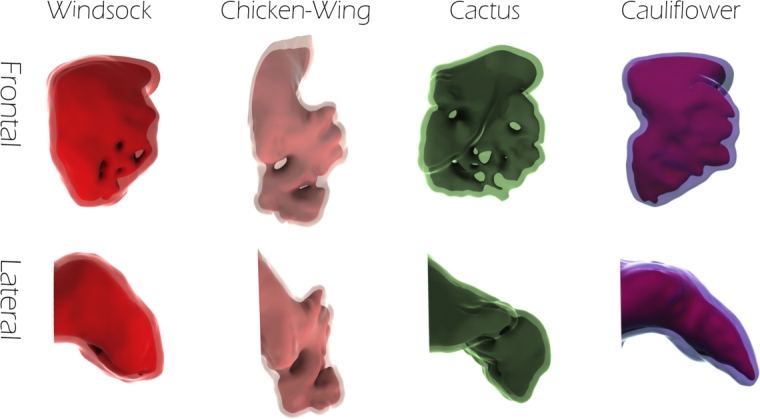
Geometric models for each morphology, the inner and the outer (in transparency) surfaces are visible. Up: frontal view; bottom: lateral view.

**Figure 2:**
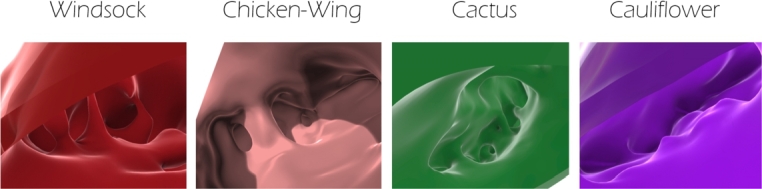
Inner trabeculae are visible for each LAA model.

The geometric models were obtained from the 3D anatomical shapes of LAA reconstructed by computerised tomography (CT) clinical exams of four patients, each one belonging to a different morphological class, namely: chicken wing, cactus, windsock and cauliflower [32], [34]. Patients were considered as control subjects, since they were not affected by known atrial pathology. The CT images were collected at University College London Hospital (London, UK). A procedure was implemented to obtain a computer-aided design (CAD) model of each class (see Fig. 1), preserving the complexity of the LAA inner morphologies, characterised by the presence of bulges and trabeculae, as shown in Fig. 2. The inner surface of the LAA were reconstructed from CT scans with slice thickness of 0.625 mm and in-plane resolution of 0.488 mm; using the Mimics package (Materialise NV, Leuven, Belgium) [32]. In detail, a manual threshold was applied to define a 2D mask of the region of interest according to the grey values highlighted by the contrast medium, followed by a cropping and a region growing algorithm, to eliminate disconnected parts. Some manual editing was then necessary to ensure that all observable anatomical structures were identified in the segmentation. The resulting 3D virtual object was finally smoothened and re-meshed, to avoid the presence of faulty mesh triangles.

The outer surface was obtained from the inner one using the commercial software Autodesk MeshMixer [35], removing the holes due to the presence of trabeculae, smoothing the surface and offsetting it externally by 2.1 mm, which corresponds to the average thickness of the LAA wall measured in ovine models [36]. Successively, the commercial software Rhinoceros 7 was used to couple the two surfaces and enclose the heart tissue of the LAA. In Fig. 1, the outer surface is shown in transparency, so as to make also the inner surface visible. Fig. 2 shows the various types of trabecular irregularities observed in the different morphologies, consisting in cords of tissue connecting the facing sides of the appendage walls so as to create a labyrinth pattern. Particular attention was paid to the modelling of the inner trabeculae, since these structures may play a role in reducing the risk of LAA inversion, by acting as ties connectors between two opposite walls of the appendage and opposing their expansion during the curvature reversal. Worth mentioning, while the inner cavity of the chicken wing, cactus and windsock models contains numerous trabeculae, the cauliflower morphology presents some structures similar to trabeculae but different, resembling septi formed by a wall thickening.

The *Abaqus CAE* (*Simulia, Dassault Systems*, Providence, RI, USA) commercial package was used to complete the computational models and to perform the numerical simulations with double precision. The four models were meshed with tetrahedral elements of the first order, with global size selected from convergence analysis conducted on the chicken wing morphology, which is reported to be the most common one, and included complex features such as lobes and trabeculations. Three meshes were generated with 6399, 8205 and 9785 elements, respectively, so as to have 20% element number refinement between each mesh and the next. The two finer meshes produce the same inversion dynamics and almost identical results (variations below 3%) in terms of stresses at the regions of higher load (the trabeculae), indicating that the mesh refinement of the model discretised into 8205 elements is adequate to describe the process. This density was used for all models, leading respectively to 8291 elements for the cactus model, 9244 elements for the windsock model, and 9182 elements for the cauliflower model. Average edge length was variable among the models, ranging from 1 to 1.5 mm, with the shortest edge between 0.1 and 0.4 mm.

The material of the LAA cardiac tissue was modelled as nearly incompressible isotropic hyperelastic, using Ogden's formulation [37], [38], [39]. This models the mechanical response on the basis of the temperature-dependent material parameters $\mu_{i}$, $\alpha_{i}$ and $D_{i}$, where parameters $\alpha_{i}$ define the nonlinearity of the stress-strain response, $\mu_{i}$ are associated with the shear modulus and $D_{i}$ with the bulk modulus. Coefficients $\mu_{i}$ and $\alpha_{i}$ were obtained by fitting the experimental data from biaxial tests conducted by De Martino et al. [40] through a nonlinear least-squares-fit procedure based on the Marquard-Levenberg algorithm [41]. $D_{i}$ coefficients were derived from $\mu_{i}$ and from the set Poisson's ratio, here imposed equal to 0.495. The associated coefficients are reported in Table 1. The material density was set to 1,120 $\text{kg}/{\text{m}}^{3}$.

**Table 1:** Coefficients for the Ogden model of the third order, *i* = 1,2,3.

	*μ*_*i*_	*α*_*i*_	*D*_*i*_
1	-0.337741884	2.00175982	9.20588005
2	0.217246844	4.00139831	0
3	0.122674830	-1.99837808	0

Bulk viscosity was included in the model by introducing the damping coefficients $b_{1}$ and $b_{2}$. $b_{1}$ damps the “ringing” in the highest element frequency, and $b_{2}$ introduces a resisting pressure that prevents the element from collapsing when exposed to high velocity gradients. The Abaqus explicit solver (with double precision) was used to perform the analyses, using an automatic time increment, with the size of the increments adjusting based on convergence and time increment estimated with the ‘element-by-element’ option. Analysis timesteps were in the order of $1\times 10^{-6}$ − $1\times 10^{-7}$ s.

**Figure 3:**
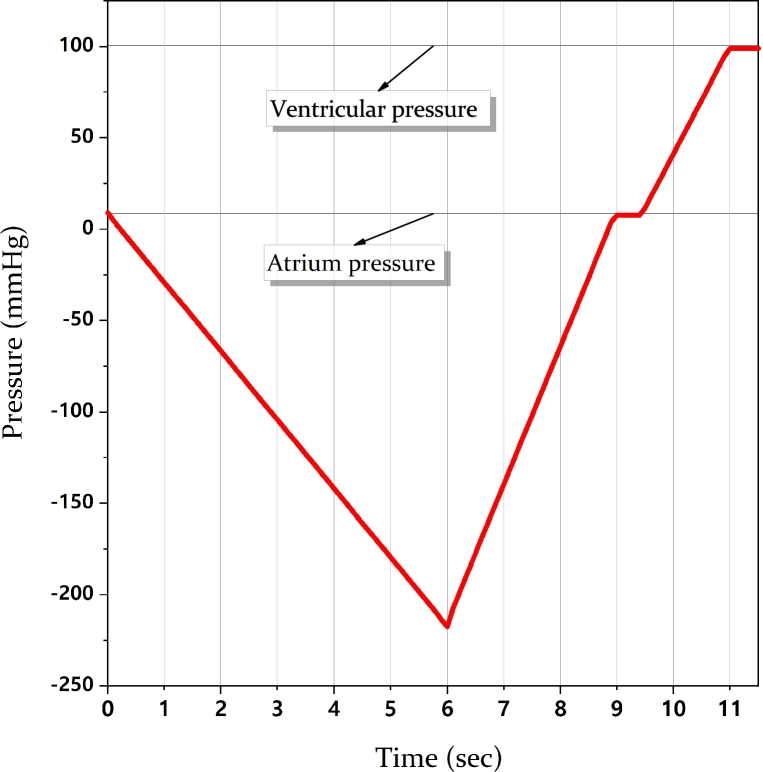
The loading pressure used for the four simulations.

The LAA inversion was simulated applying a uniformly distributed pressure to the surface of the inner cavity, varying in time; the appendage body was constrained at the orifice curve defined by the external wall, keeping it free to rotate. To investigate and compare the inversion phenomenon in the different morphologies, a pressure load curve was designed to ensure full inversion for all models. This is likely to exceed the procedural suction during de-airing maneuver, as the inversion occurs rarely. The suction required to ensure full inversion in all models, estimated by trials and errors, was equal to −225 mmHg. Then, pressure was ramped up to the mean atrial pressure (9 mmHg [42]) and, subsequently, to the systolic ventricular pressure (100 mmHg), in order to verify if the models can be drawn back to their physiological configurations by inhibiting the mitral valve closing function. The defined pressure curve involves four stages, each one used to investigate the LAA behaviour during the inversion and the recovery (see Fig. 3):

1. a negative ramp, resulting in a pressure suction of 225 mmHg (−30 kPa) in 6 s, to evaluate the appendage inversion;
2. a positive ramp to restore physiological pressure in 3 s;
3. a plateau of 0.5 s maintaining the initial pressure; in order to evaluate if returning to the physiological atrial pressure produces the recovering of the initial LAA configuration;
4. if no recovering of the initial LAA configuration is achieved at stage 3, a second positive ramp with same slope as that of stage 2, up to complete recovery of the LAA, corresponding to an increase of pressure equal to 100 mmHg (13.33 kPa - around the ventricular pressure) from the physiological initial value.

## 3 Results and discussion

**Figure 4:**
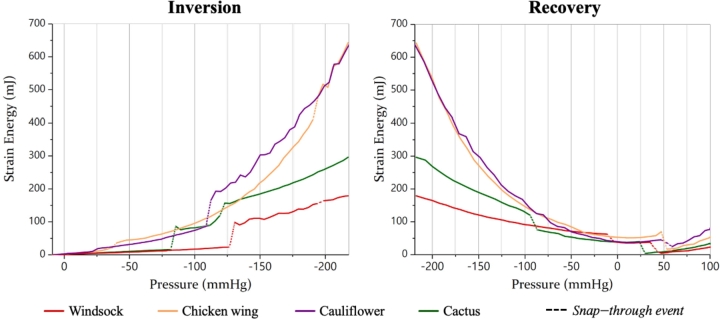
The strain energy calculated for the four simulations: on the left the inversion phase and on the right the recovery. The dashed lines indicate the energetic jumps associated with snap-through events.

From a mechanical point of view, inversion is led by two different mechanisms: eversion and snap-through. The eversion mechanism consists in the progressive streaming of the LAA wall material through the appendage orifice, by turning it inside out. This mechanism, widely analysed for flexible tubes and tubular membranes [43], [44], [45], determines a prolapse of the LAA neck into the LA chamber. This propagating mechanism is associated with a progressive increase in strain energy in the growing region that is turned inside-out and in the unturned wall contacting it. However, eversion is not *per se* sufficient to determine the inversion of the whole LAA, as the presence of trabeculae and the multiple curvatures defining the tip and lobes require a snap-through mechanism. This occurs when the equilibrium state of the system becomes unstable due to the change in loading, so the system jumps to an alternative equilibrium state, through sudden geometric modifications [46]. This phenomenon produces abrupt changes in the strain energy of the system, that can be clearly monitored during the simulation, and used for quantitative evaluation of the bio-mechanical behaviour of the appendages and of the pressures at which the snap-through events happen. In practice, the inversion occurs over a period of time, during which the LAA body crosses a series of intermediate shapes (as effect of the combination of a sequence of eversion and snap-through events). The first snap-through event results into a *initial snapped-through configuration*, which may further evolve as the eversion mechanism continues, reaching a final stable configuration associated with the final snap-through event (which depends on the presence and distribution of geometrical constraints such as trabeculations), and may still result in a partial invagination. This configuration, here indicated as *final snapped-through configuration*, keeps deforming as the acting suction increases, but does not experience observable changes in its main morphological features up to the maximum suction load. The presence of snapped-through events has also an effect on the recovery of the anatomical configuration. In fact, it results into more stable inverted shapes, which require higher energy levels to re-snap to the initial morphology. Configurations associated with the snap-through event restoring the anatomical shape during the ramps of increasing pressure will be indicated as *recovered configurations*. The snap-through events observed during inversion and recovery are represented in Fig. 4 as dashed lines.

The pressure required to cause inversion varied substantially for the different morphologies, with the *chicken wing* achieving the *initial snapped-through configuration* at the lowest suction, −43 mmHg, and the others at much higher negative pressures (−88 mmHg for the *cactus*, −111 mmHg for the *cauliflower* and −133 mmHg for the *windsock*) (see Fig. 4). These pressures are substantially larger than the typical suction during de-airing, which is expected to be below 30 mmHg [47], and would not produce inversion in any of the analysed models. However, the *chicken wing* would not require a much larger suction excess, confirming that there are LAA shapes more at risk than others.

The strain energy stored to achieve this *initial snapped-through configuration* is different for the four cases, and not directly related with the corresponding pressure. The *cactus* inverted at the minimum value, 16 mJ, the *chicken wing* at 21 mJ, the *windsock* at 25 mJ and the *cauliflower* at 86 mJ. As the pressure rises to 30 kPa, the *chicken wing* and *cauliflower* experience the largest increase in strain energy, reaching 643 mJ and 637 mJ respectively. At the same load, the *windsock* has a strain energy value equal to just 179 mJ (see Fig. 4).

Recovery required some excess of pressure which, as for the inversion, was largely different for the various configurations. The *cactus* required an excess of 31 mmHg, the *windsock* of 39 mmHg, and the *chicken wing* and *cauliflower* of 54 mmHg (see Fig. 4). It is worth noting that the pressure requested to restore the *chicken wing* anatomical configuration is similar and higher than the suction magnitude required for its inversion.

**Figure 5:**
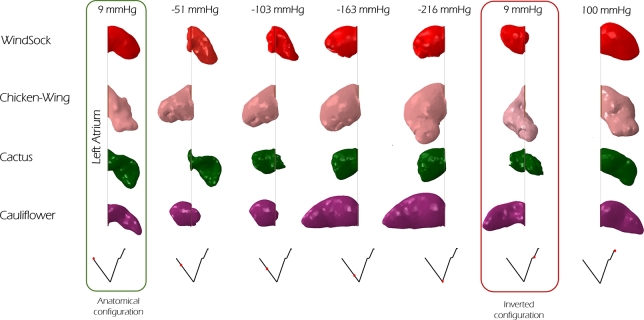
Successive snap-shots taken by four models during the loading pressure evolution. On the top, the corresponding pressures; on the bottom, the pressure evolving in time.

A sequence of snap-shots that each morphology takes during the loading pressure evolution (see Fig. 3) is illustrated in Fig. 5. Interestingly, none of the inverted configurations spontaneously recovers the anatomical shape when restoring the initial pressure (9 mmHg), but they all maintained a reverted or semi-reverted configuration (see Fig. 5).

**Figure 6:**
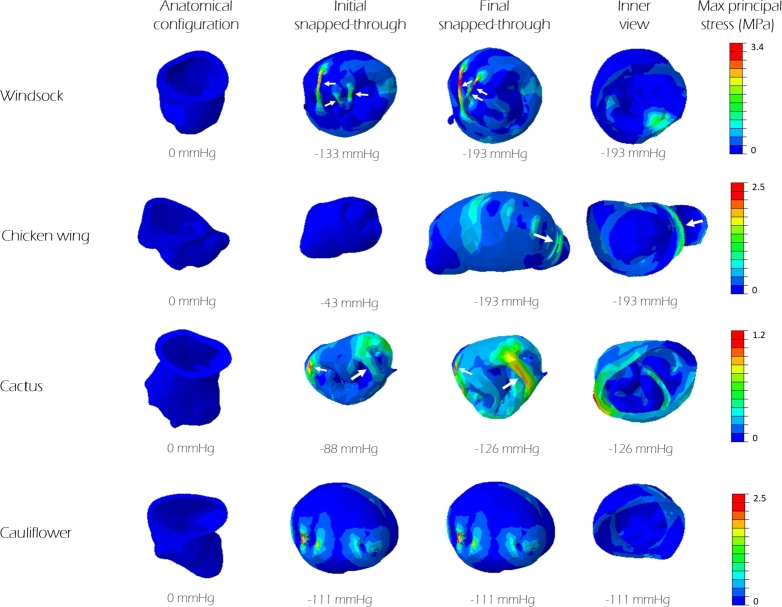
For each morphological class, the columns indicate the geometries corresponding to the *anatomical configuration*, to the *initial snapped-through configuration* and to the *final snapped-through configuration*. The visible trabeculae are indicated by white arrows. The corresponding pressure is reported at bottom of each configuration. On the right, a view of the inner cavity (*inner view*) of the LAA allows to observe the tip at the *final snapped-through configuration*. Colours represent the magnitude of maximum principal stress.

The *initial* and *final snapped-through configurations* obtained for each model are shown in Fig. 6.

In the case of the *cauliflower*, a single snap-through event is observed, and therefore the *initial* and *final snapped-through configurations* coincide. In this model, in fact, the regularity of the LAA shape (where lobes and trabeculae are not significant) allows eversion as the dominant mechanism up to the final stage, which is completed with the snapping of the tip (see Fig. 6).

In fact, the *initial snapped-through configuration* displayed in Fig. 6 suggests that trabeculae limit the tip inversion, requiring further snap-through events at higher pressures (for *windsock* and *chicken wing* a pressure of −193 mmHg is requested). For all models, the *final snapped-through configuration* is characterised by the LAA distal tip elongated into the atrial chamber, except for the *cactus*, where the tip remains invaginated into the LAA walls. Moreover, this model is the first to recover its natural configuration (at the lowest pressure), as evident from the diagram of the recovery in Fig. 4.

Fig. 6 shows the max principal stress estimated in the different LAA models at the *initial* and *final snapped-through configurations* of the inversion process. Maximum values are concentrated at the trabeculae, that stretch during the process, acting as tie beams. The stress becomes maximum, reaching about 3.5 MPa, in the *windsock* model, where some trabeculae have small cross section (diameter 0.8 mm). The level of stress reduces substantially in the *chicken wing* and *cactus* models, where trabeculae have larger cross sections (diameter between 2.0 mm and 4.3 mm). In the case of the *cactus*, which is characterised by a large number of thick trabecular structures spread between the medial and lateral wall of the appendage, these are sufficient to prevent the tip elongation into the atrial chamber, which stays invaginated also at the highest suction pressure. In the case of the *cauliflower* model, which does not present trabeculae in the form of columnar protrusions connecting the walls of the chamber, it is evident from the stress distribution in Fig. 6 that tie structures with the same function are present, although they are embedded in the wall, as mentioned in Section 2. Apart from the trabecular structures, the body keeps low values of stress, below 1 MPa for all models.

In summary, of the analysed morphologies, the *chicken wing* required the lowest suction pressure to achieve inversion, and the highest pressure to recover the anatomical configuration. On the contrary, the *cactus* model resulted the safest one, being the only one where the tip remained invaginated and never fully protruded into the atrium (see Fig. 6, Inner view), and recovering the anatomical configuration at the smallest pressure (see Fig. 4). In terms of inverted shape, as shown in Fig. 5, the *chicken wing* and *cauliflower* models resulted into longer protrusions into the atrium, very angled in the case of the *chicken wing*. This may be associated with higher risk of interference with the pulmonary veins access or with the mitral valve inlet. This is a reported complication of LAA inversion, resulting in right ventricular failure due to pulmonary hypertension and elevated right-sided filling pressures [6]. This risk appears to be reduced with highly trabeculated morphologies such as, in the analysed cases, the *cactus* and the *windsock* models, that invert partially or in a more compact configuration. Importantly, all models recovered the anatomical configuration at pressures well below the ventricular pressure (about 100 mmHg). This suggests that basic trans-catheter manoeuvres, e.g. producing temporary mitral regurgitation, could be attempted to verify the presence of LAA inversion and correct it, prior to proceed with more invasive approaches.

The models include a number of approximations that may affect the results. In fact, although they reproduce faithfully the inner wall surface of the LAA structure, the average wall thickness and the material properties were assumed identical between models, and based on studies performed on animals. In order to maintain the computational cost acceptable, only the LAA structure was modelled, thus requiring the introduction of assumptions on the boundary conditions. In particular, nodes at the outer edge of the attachment section between the appendage and the main atrial chamber were constrained in position, and left free to rotate. As the wall thickness of the atrium is comparable to that of the appendage [48], neglecting its mechanical resistance may result in some overestimation of the appendage deformation. On the other hand, the presence of a constraint preventing the enlargement of the orifice during deformation may have the opposite effect. Also, to preserve the patient specific shape of the appendage, which is acquired during operating conditions, the stress distribution caused by the physiological pressure was neglected. The assumptions described in this study were not investigated in terms of quantitative impact. In fact, the aim of this work is not to quantify the risk for a specific patient, but to investigate the mechanisms responsible for this rare complication and their potential dependency on the individual LAA morphology. To transfer the proposed approach to clinical applications, further investigations aimed at quantifying the effect of variabilities in the thickness, material model and boundary conditions would be required. Still, this first study clearly indicates that patient specific morphological and topological features of the LAA play a crucial role on the phenomenon, and this information can be used to identify the patients for which the risk of LAA inversion should be considered. A further limitation of this computational model is the absence of validation with clinical data which, due to the rarity of this phenomenon, are not available in the literature. However, more attention should be directed towards this phenomenon, as its poor knowledge makes it often mistaken for a clot formation or a mass [6], [8], [11], [14], [15], [17], [18], [19], [20], [23], [25], with serious clinical implications. Moreover, the understanding of the involved mechanisms is now gaining increasing significance, with LAA inversion recently proposed as a potential therapy to prevent clot formation in atrial fibrillation patients [29], [30], [31].

## 4 Conclusion

The presented study allowed to predict and compare the risk of LAA inversion for different patient specific anatomies, each belonging to a different LAA morphological class. The analysis indicates that, in dependence of the anatomy, the pressure required to cause inversion and recovery of the appendage varies substantially, suggesting that the risk of inversion is strongly associated with the specific LAA morphology. For all analysed cases, the physiological left atrial pressure appeared to be insufficient to restore the correct anatomical LAA configuration, although manoeuvres exploiting the left ventricular pressure (e.g. by forcing temporary mitral valve regurgitation) could be helpful in restoring the LAA natural configuration. The analysis clearly indicates that patient specific morphologies, such as the *chicken wing* model in the presented study, can be very critical in terms of risk of inversion, inability to recover spontaneously their correct anatomical configuration and potential interference with the pulmonary vein access or mitral inflow. Others, such as the *cactus* model in the study, appear to be particularly safe. However, it is crucial to clarify that, as the results are single cases, they shall not be considered representative of the whole morphological classes, and a number of parameters not directly associated with the classification appears to play a crucial role on the phenomenon, such as the amount and location of the trabecular structures. Further studies on wider data-set would be needed to investigate association between LAA inversion and the morphological parameters that control it.

## Ethics statement

This study was carried out in accordance with the recommendations of the South East Research Ethics Research Committee, Ayelsford, Kent, United Kingdom. All patients/participants participate in this study in accordance with the Declaration of Helsinki. The protocol was approved by the South East Research Ethics Research Committee, Ayelsford, Kent, United Kingdom.

## CRediT authorship contribution statement

**Danila Vella:** Conceptualization, Data curation, Formal analysis, Investigation, Methodology, Supervision, Validation, Visualization, Writing – original draft. **Giulio Musotto:** Data curation, Formal analysis, Investigation, Methodology, Validation, Visualization, Writing – review & editing. **Andrew Cook:** Data curation, Formal analysis, Investigation, Resources, Writing – review & editing. **Giorgia Maria Bosi:** Formal analysis, Investigation, Methodology, Supervision, Validation, Writing – review & editing. **Gaetano Burriesci:** Conceptualization, Data curation, Formal analysis, Funding acquisition, Investigation, Methodology, Project administration, Resources, Supervision, Validation, Writing – review & editing.

## Declaration of Competing Interest

The authors declare that they have no known competing financial interests or personal relationships that could have appeared to influence the work reported in this paper.

## Data Availability

The data that support the findings of this study are available from the corresponding author upon reasonable request.
